# The correlation between obesity and the occurrence and development of breast cancer

**DOI:** 10.1186/s40001-025-02659-4

**Published:** 2025-05-26

**Authors:** Jun-Jie Hu, Qi-Yue Zhang, Zhi-Chun Yang

**Affiliations:** 1 Hunan University of Traditional Chinese Medicine, Changsha, 410078 China; 2 Department of Pharmacology, Xiangya School of Pharmaceutical Sciences, Central South University, Changsha, 410078 China

**Keywords:** Obesity, Adipokine, Adipocyte, Tumour microenvironment, Breast cancer, Metabolic disorders, Inflammatory

## Abstract

This study reviews the mechanisms by which obesity affects the development and progression of breast cancer (BC). The association between obesity and BC is mainly due to three aspects: disruption of glycolipid metabolism, abnormal cell function and imbalance of adipokine levels. The dysregulation of glycolipid metabolism caused by obesity, including the accumulation of cholesterol and fatty acids and the reprogramming of glucose metabolism, promotes the growth and invasion of tumour cells. Obesity triggers multiple cellular abnormalities, particularly in lipid-associated macrophages and cancer-associated adipocytes, which promote tumour progression and immunosuppression by secreting inflammatory factors and various fatty acids into the tumour microenvironment. Obesity leads to an imbalance in the expression of several adipokines. Leptin upregulation is closely associated with BC metastasis and resistance to endocrine therapy, while reduced adiponectin levels attenuate the protective effect. At the same time, chronic inflammation and insulin resistance not only further increase the risk of BC, but also exacerbate tumour resistance. In terms of treatment, weight-loss drugs and metformin can improve the efficacy of obesity-related BC treatment to some extent. Intervention strategies targeting adipose tissue remodelling, lipid metabolism and leptin regulation also show potential clinical value, but more research is needed to clarify their safety and efficacy. This review provides systematic ideas and references for research into the mechanisms and clinical management of obesity-related BC.

**Graphical abstract:**

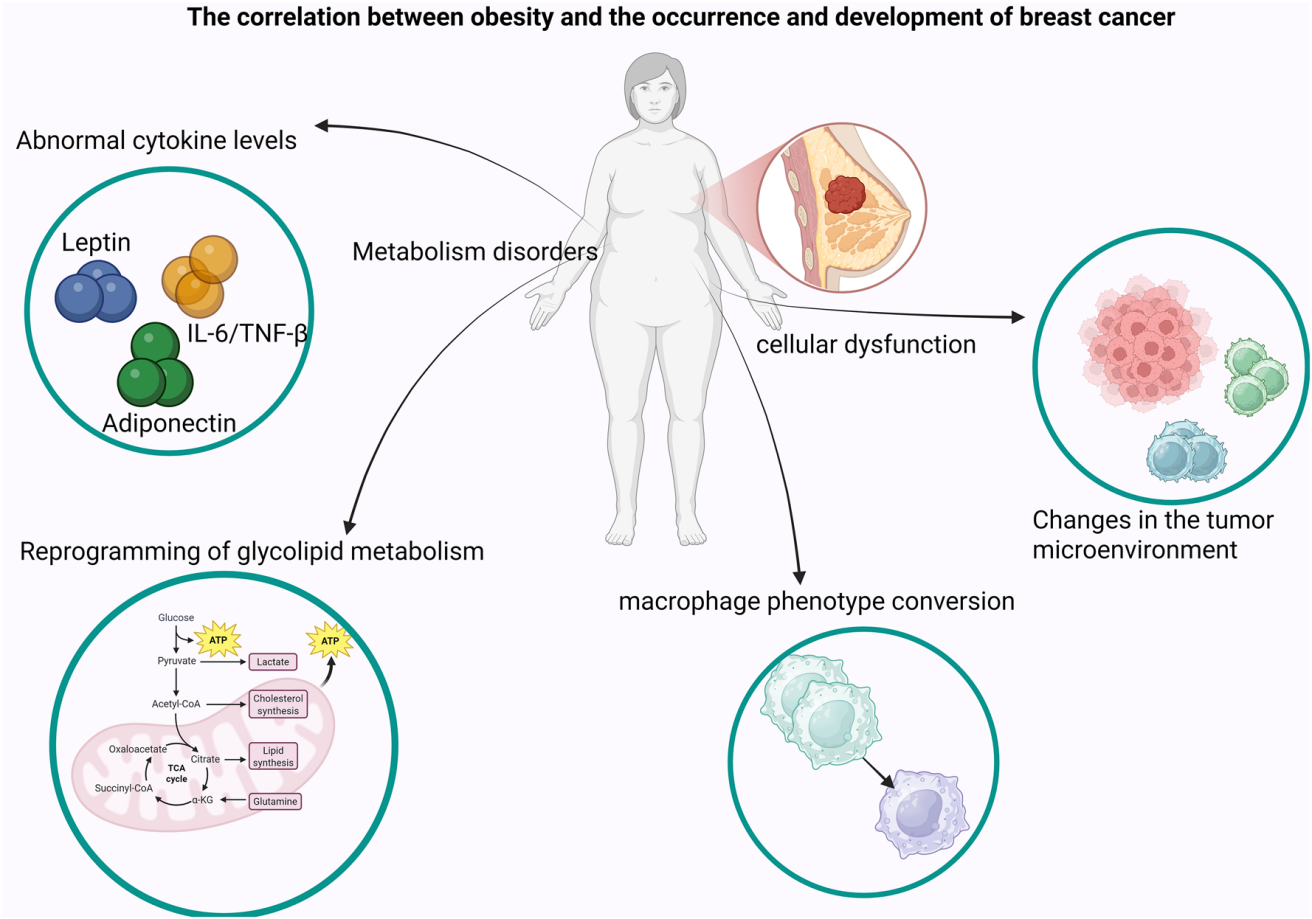

## Introduction

BC has emerged as a significant global health problem [1]. Studies indicated a close correlation between obesity and BC, one consequence of obesity leading to an increased rate of BC malignancy is triple-negative breast cancer (TNBC) [2, 3], which garnered considerable interest of researchers. This article summarises relevant literature based on the existing databases Web of Science and PubMed to explain the link between obesity and BC.

There is a certain correlation between BC and genes related to obesity. The association between single nucleotide polymorphisms and BMI was assessed using a linear regression model for 1561 candidate obesity-related genes in the study by Chung, S. et al. analyzing the association between obesity-related genes and BC risk. Among the 890 genes, 12,388 single-nucleotide polymorphisms (SNPs) were associated with BMI (P < 0.05). The most significantly associated SNPs with BMI were rs17804012 in *RBFOX1* and rs2014791 in *LINC00317* (P < 5E–6), yet these SNPs exhibited a zero correlation with BC risk. Conversely, genes with a lower correlation with BMI (N = 644, P < 0.05) exhibited a significant correlation with BC risk (the pathway P < 0.003). The effect of the *THRB* gene on BC risk is of particular significance (P = 9E–04), and is confined to premenopausal women with a BMI above 23.2 (P < 0.01). The BMI-related gene set, which includes *THRB* in the pathway analysis, exhibits a significant association with BC risk and varies depending on menopausal status and/or BMI level [4]. Concurrently, in the study by da Cunha, PA et al., an analysis of 100 BC patients and 148 healthy women from Santa Catarina, Brazil, found that the interaction between Obesity Associated gene (*FTO)* and melanocortin 4 receptor gene (*MC4R)* polymorphisms is strongly associated with BC. Women who possessed the allele combination C/T/C (*FTO rs1121980*/*FTO rs9939609*/*MC4R rs17782313*) exhibited a 4.59 times higher risk (P = 0.0011, adjusted for age and BMI). These associations did not appear to be associated with BMI, suggesting that the polymorphisms in *FTO* and *MC4R* directly influence BC risk [5]. The aforementioned studies have demonstrated a correlation between obesity and an elevated risk of developing BC [5]. In another study of BC risk in obese women, Neuhouser, ML et al. noted that overweight and obese women have a higher risk of BC than normal-weight women. A BMI of 35.0 or higher is strongly associated with the risk of estrogen receptor-positive(ER +) and progesterone receptor-positive(PR +) BC, but not with estrogen receptor-negative BC [6]. Recent research has elucidated the role of adipokines in the development of BC. In studies by Gui et al. subjects with a BMI > 25 kg/m^2^ exhibited significantly lower adiponectin levels and higher leptin and tumour necrosis factor-α (TNF-α) levels compared to subjects with a BMI < 25 kg/m^2^. The study's findings indicate that decreased concentrations of adiponectin and increased concentrations of leptin, Interleukin-6(IL-6), Interleukin-8(IL-8), TNF-a, resistin, and visfatin are significantly associated with an elevated risk of developing BC [7]. It has been demonstrated that these adipokines are capable of regulating cancer metabolism and activating inflammatory pathways. The inflammatory response is primarily attributed to the recruitment and activation of inflammatory mediators by macrophages. The chronic inflammatory state associated with obesity is further exacerbated by macrophages, contributing to the development of the tumour microenvironment [8, 9]. Obesity has also been reported to promote BC metastasis by affecting the microenvironment. In a mice experiment, the researchers gave mice a regular diet and a high-fat diet and observed the two groups by transplanting tumour cells. The results showed that the pro-metastatic effect of obesity is produced by affecting tumour cells in the primary tumour [10].

It is worth noting that when we talk about BC, we usually think of female BC, but in fact male BC is gradually coming into the public eye. Male BC is rare, accounting for about 1% of male cancers and 1% of all BCs worldwide [11]. As the causes and pathogenesis of male BC are unclear, the current treatment strategy for male BC is mainly based on the recommendations for female BC. At the same time, researchers have mentioned that, as this is a stigmatising diagnosis for men, appropriate psychological treatment is needed [12].

In light of these considerations, understanding the specific mechanisms by which obesity affects BC is critical to manage the disease in obese women. This review aims to elucidate the links between obesity and BC, focusing on metabolic dysregulation, adipokine imbalance and inflammation as key factors.

## Epidemiologic evidence on breast cancer and obesity

The definitions of the molecular subtypes of BC were presented at the 2013 St. Gallen International Breast Cancer Conference, namely, luminal A (ER/PR positive, HER2 negative, KI67 < 20%, the percentage indicates the results of immunohistochemical staining of patient samples), luminal B is divided into HER2 negative (ER/PR positive, ER/PR positive, HER2 overexpression), Erb-B2 overexpression (HER2 overexpression, ER/PR absent), basal-like TNBC (ER/PR absent, HER2 negative) and other specific subtypes [13].

Epidemiological evidence supports the association between obesity and an increased incidence of BC. Statistics reveal that obese postmenopausal women face a significant rise in the risk of developing invasive BC. A BMI of over 30 is considered to be obese, while a BMI of between 25 and 30 is considered to be overweight [14]. Compared with women with a normal BMI, obese grade 1 (BMI ≤ 35 kg/m^2^) and obese grade 2 + 3 (BMI > 35 kg/m^2^) women had an increased risk of ER + /PR + BC by 52% and 86%, respectively, suggesting that obesity increases the risk of BC significantly in obese women, especially in obese grade 2 + 3 women [6]. Obesity has a particularly serious impact on African–American women. In a case study of 1891 cases, higher levels of obesity, especially higher waist-to-hip ratios, were associated with poorer survival. Central obesity and high waist-to-hip ratios may be the main reasons for the poorer BC prognosis in African American women [15]. Furthermore, the impact of obesity on BC incidence among Mexican women highlighted the potential role of metabolic disorders and various contributing factors in the etiology of BC, among which obesity-induced inflammation and metabolic dysregulation were important factors in BC progression [16]. Obesity is independently associated with increased BC risk in postmenopausal East Asian women (obesity is defined in this article as a BMI ≥ 25), and metabolically unhealthy women also have an increased risk of BC, according to Park et al. [17]. There are two main reasons for this racial difference. One is that African Americans are more affected by central obesity [18]. The other is metabolic changes. For example, East Asian women are more affected by postmenopausal metabolic syndrome, which is associated with an increased risk of BC [17]. Obesity-induced fatty liver also increases the risk of BC. In postmenopausal women, a Fatty liver index (FLI) of 60 or higher was significantly associated with an increased risk of BC [19]. Obese women also have a higher risk of distant metastasis and death from BC. Patients with a BMI of 30 kg/m^2^ or higher have a significantly increased risk of distant metastasis by 46% after 10 years and a significantly increased risk of death from BC by 38% after 30 years. However, BMI has no effect on the risk of local recurrence [20].

However, the link between obesity and BC in pre-menopausal women is unclear. For example, in reports such as Crispo et al. central obesity significantly increases BC mortality in pre-menopausal women [21]. Obesity has a protective effect against BC in pre-menopausal women in Europe, whereas obese postmenopausal women have an increased risk of BC, according to a systematic review and meta-analysis [22]. Therefore, more epidemiological evidence is needed on the association between obesity and BC risk in pre-menopausal women.

## The link of breast cancer and obesity

### Obesity-induced-metabolic disorders and breast cancer

**Fig. 1:**
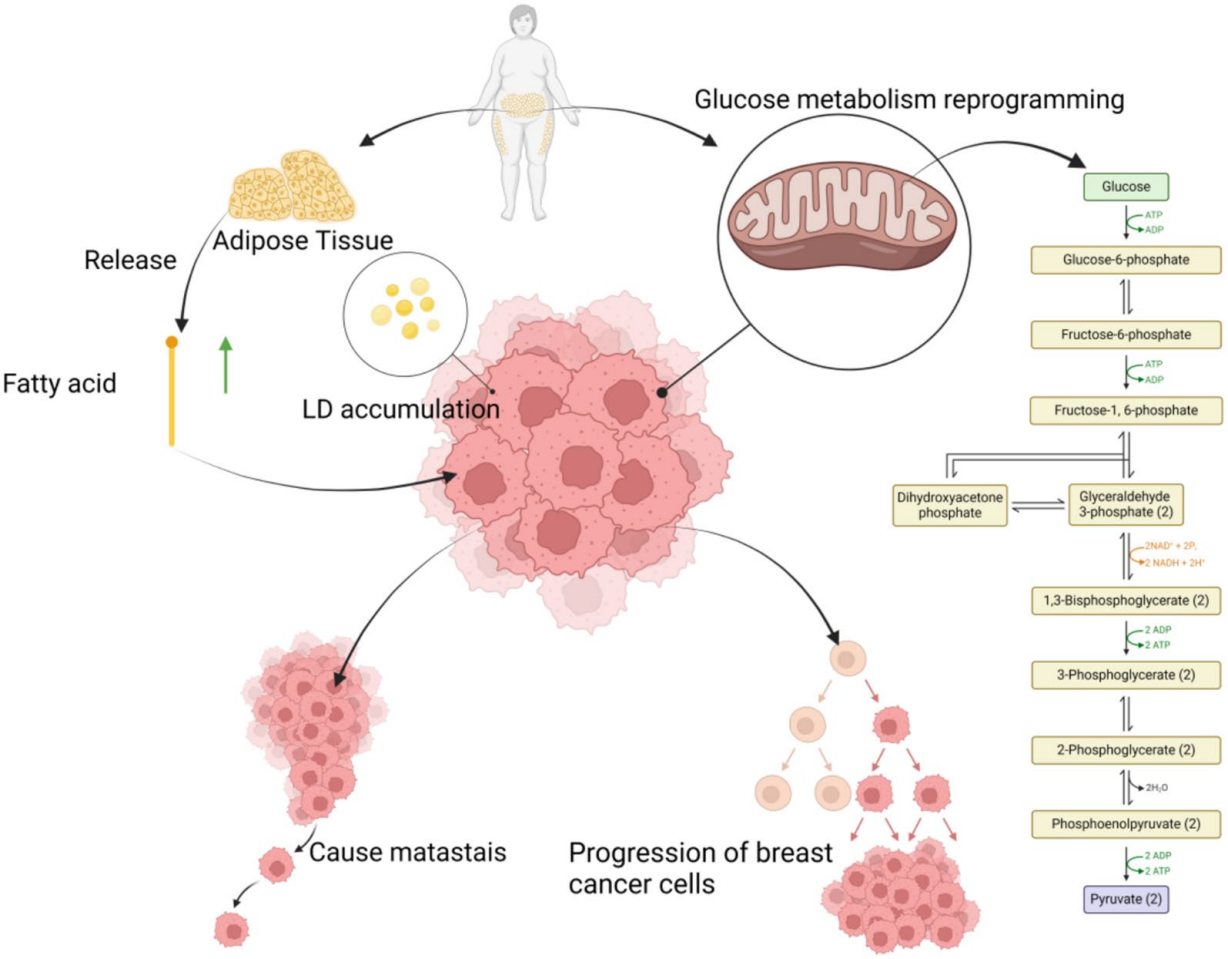
Obesity-induced-metabolic disorders and breast cancer

Obesity-induced alterations in glycolipid metabolism have been proved to be associated with the progression of BC. The primary mechanism of action is reprogramming of glucose metabolism and lipid accumulation in BC. The reprogramming of glucose metabolism constitutes a significant hallmark of cancer cell transformation. The accumulation of lipids in cells greatly promotes the proliferation and invasion of BC cells [23] (Fig. 1). Extracellular, the lipid metabolism disorder engendered by obesity is characterised by elevated levels of cholesterol, triglycerides, and low-density lipoprotein (LDL). Current research indicates that the accumulation of lipids is generally deleterious to human health, particularly the elevated levels of extracellular cholesterol and fatty acids, which are considered to be pivotal factors in the promotion of BC proliferation and metastasis [24, 25]. The mechanism by which fatty acids promote BC proliferation and metastasis is mainly CD36-dependent. They specifically enhance the metastatic potential of CD36-positive metastasis-initiating cells [26].

This figure shows that free fatty acids released from adipose tissue are taken up by BC cells, where they form lipid droplets (LDs) inside the cell. This, combined with the reprogramming of glucose metabolic pathways, provides the tumour with additional energy and metabolic intermediates, thereby promoting the proliferation and metastasis of BC cells. It also briefly illustrates the key steps of glycolysis and its synergistic effect with lipid accumulation on the malignant progression of BC.

#### Cholesterol metabolism

Reprogramming of cholesterol metabolism is a hallmark feature of tumour cells, which require high levels of cholesterol for membrane biogenesis and other functional requirements [27]. Thus high cholesterol due to obesity promotes the progression of cancer cells. Studies have shown that cholesterol synthesis plays a critical role in the proliferation of BC stem cells, which are key drivers of tumour growth and metastasis [28]. The activation of the cholesterol biosynthesis pathway has been identified as a significant characteristic associated with the increase of tumour stem cells in TNBC [29]. Their findings further indicate that inhibiting cholesterol biosynthesis pathway in tumour stem cells leads to a reduction in mammosphere formation and growth, highlighting cholesterol as a potential therapeutic target in BC treatment, particularly in strategies aimed at tumour stem cell therapy [28]. Recent research has revealed that a long non-coding RNA named lncRNA30, which facilitates the elevation of cholesterol, is crucial for maintaining the stemness of BC stem cell through activation of PI3K/AKT pathway [30]. NAD/NADP dependent steroid dehydrogenase-like (NSDHL), a crucial enzyme in cholesterol metabolism. In the study by Chen et al. NSDHL was identified as a potential metastatic driver in TNBC. The study revealed that NSDHL exhibited elevated levels of expression in BC tissues, and that the suppression of NSDHL significantly impeded cell proliferation and migration. The study further elucidates the pivotal role of NSDHL in mediating metastasis through the NSDHL–TGF β R2 pathway [31].

In the context of patients with obese BC, cholesterol has been reported to facilitate the proliferation of cancerous cells by counteracting oxidative stress. This process involves regulating the synthesis of NADPH and the elimination of Reactive Oxygen Species (ROS), in addition to the activation of human estrogen-related receptor α (ERRα). These studies suggest a distinct role for cholesterol in the development of BC [32, 33]. However, the molecular processes by which cholesterol impacts BC, along with interventions aimed at modulating cholesterol levels for therapeutic benefit, should be highlighted and given priority in future research.

#### Fatty acid metabolism

**Fig. 2:**
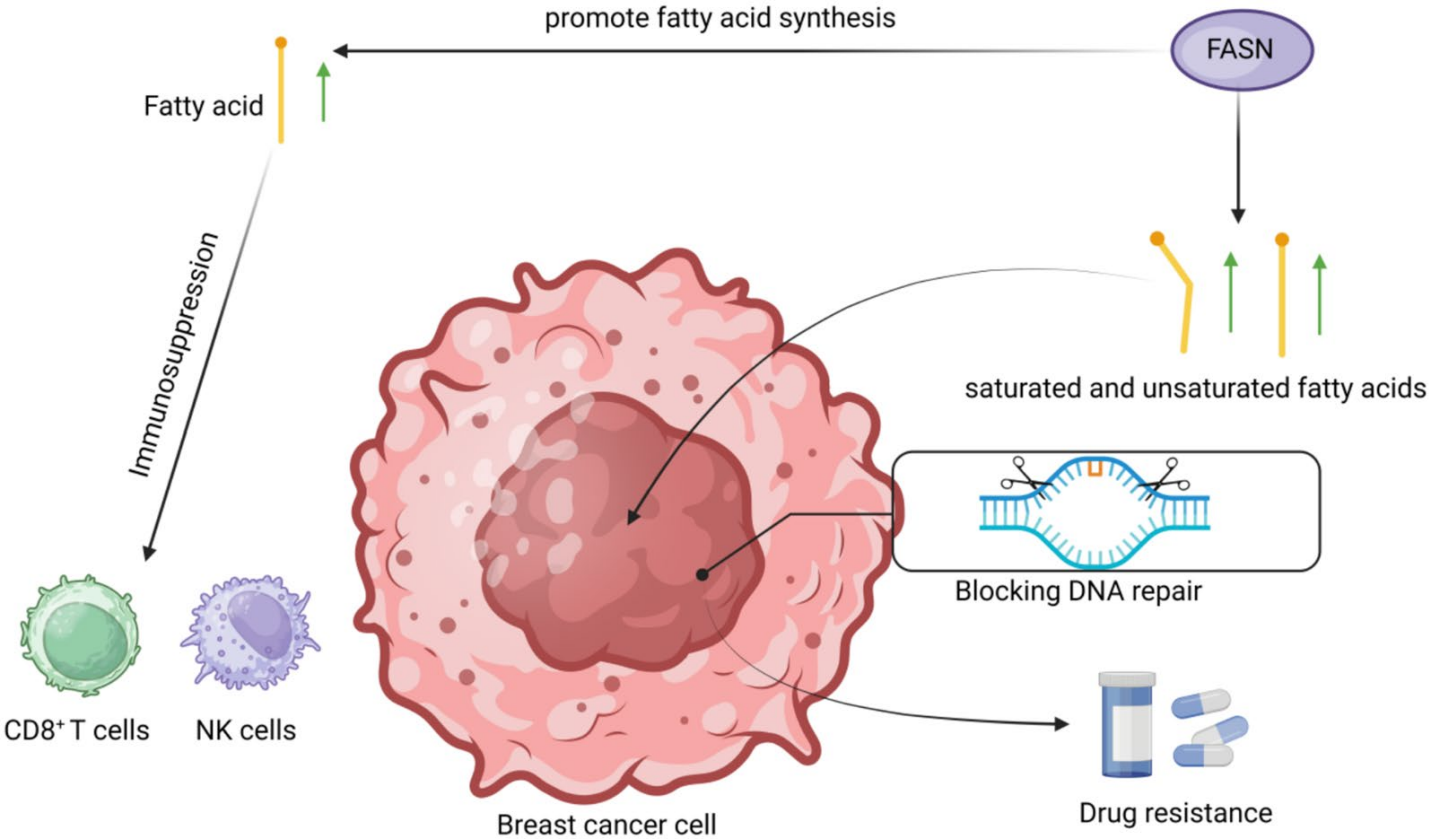
Role of FASN in breast cancer cells

Elevated levels of fatty acids, frequently observed in BC patients, persistently remain abnormal even after surgical interventions, this suggests that fatty acids may be influencing factors in the development of BC rather than a product of BC [34]. The dysregulation of de novo lipid biosynthesis and the uptake of exogenous FA not only helps cancer cells to maintain their rapid rate of proliferation, but also provides them with an important source of energy under conditions of metabolic stress [35]. The key enzyme fatty acid synthase (FASN), which is involved in the de novo synthesis of fatty acids, is overexpressed in tumour tissues. In normal tissues, it is either not highly expressed or only weakly expressed [36]. In addition to FASN, acetyl-CoA carboxylase-1 in the de novo fatty acid synthesis pathway is also involved in reprogramming lipid metabolism. ACC1 can confer metabolic adaptability to drug-resistant tumours by promoting the enrichment and active function of lipid droplets and peroxisomes [37]. Under normal conditions, cell proliferation requires fatty acids for the synthesis of membranes and signalling molecules, whereas in cancer cells the fatty acid requirement rises considerably and affects part of the signalling pathway [38]. A strong correlation between fatty acid accumulation and the malignancy of BC has been established in current research. The synthesis of fatty acids is crucial for the invasion of BC into the brain [39]. The secretion of fatty acids into tumour microenvironment significantly affects the infiltration of immune cells, predominantly by diminishing the activity of CD8^+^ T cells and impacting both the activity and functionality of NK cells [40] (Fig. 2).

The fatty acid de novo synthesis pathway plays an important role in the progression of cancer cells. Most cancer cells rely on fatty acid synthesis mediated by FASN to maintain the demand for cell growth [41]. In addition, research suggests that fatty acids synthesised through FASN-mediated synthesis may be a factor in promoting BC metastasis and growth, FASN is found to be elevated in 30% of Human Epidermal Growth Factor Receptor 2 (HER2)-overexpressing BC [42]. Xu et al. reported that the serum of patients with invasive ductal carcinoma had elevated levels of 12 types of fatty acids. Whereas, after reducing the expression level of FASN, intracellular unsaturated fatty acids and saturated fatty acids were reduced, and cell migration was inhibited, the results of this study suggest that FASN may be influencing the progression of BC cells by altering the levels of specific fatty acids [43] (Fig. 2). Therefore, targeting against FASN with or without other molecular targeting drugs has demonstrated certain anti-tumour effect [44]. Although the role of FASN in BC cells has been widely discussed, treatment via FASN inhibitors has not made great strides due to the side effects of FASN inhibitors.

This figure shows that obesity promotes the excessive synthesis of saturated and unsaturated fatty acids by upregulating the expression of fatty acid synthase (FASN). Excess fatty acids not only weaken the ability of CD8⁺ T cells and NK cells to eliminate tumour cells through immunosuppression, but also interfere with DNA repair processes, leading to the development of drug resistance and continued tumour progression. The arrows, symbols and text in the figure show that fatty acid synthesis and immunosuppression play an important role in the development of drug resistance and malignant phenotypes in BC cells.

Furthermore, the expression of very long-chain fatty acid protein 5 (Elovl5) has been identified as a contributing factor to BC metastasis by up-regulating transforming growth factor-β (TGF-β) receptor via the promotion of lipid droplet (LD)-accumulation-dependent Smad2 acetylation. A notable finding was the observation that Elovl5 mRNA levels were found to be higher in ER + BC cases compared to those observed in HER2 + positive and TNBC cases. Thus, it might be a predictive biomarker for metastatic ER^+^ BC [45]. Since neutral lipid accumulation in BC cells significantly contributes to both the proliferation and metastasis of the disease. Strategies aimed at blocking neutral lipid accumulation may offer a novel therapeutic approach in treating BC.

#### Glucose metabolism

**Fig. 3:**
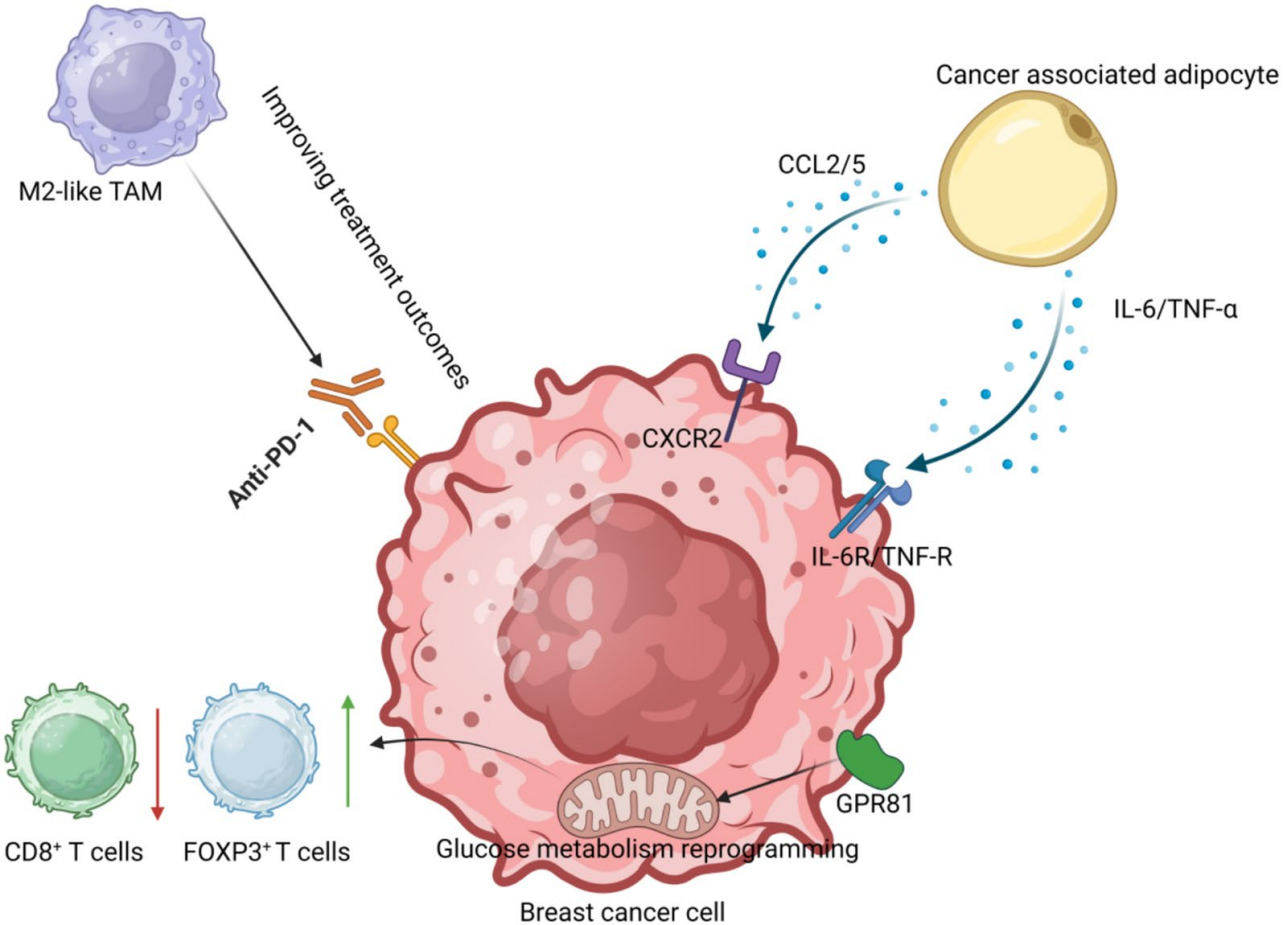
Detailed explanation of specific mechanisms in TAME

It is a common trait of tumour cells that predominantly utilises glycolysis for energy production. Independent association between hyperglycemia and luminal BC has been observed [46]. Furthermore, elevated glucose levels were found to stimulate the proliferation of MCF-7 BC cells [47]. Studies have shown that rats with impaired glucose tolerance exhibit a higher risk of BC, attributed to the IGF-1/PI3K/Akt/GSK3β/β-catenin signalling pathway [48]. Insulin resistance is a complex metabolic disorder that cannot be explained by a single aetiological pathway. Its main manifestation is reduced sensitivity to insulin [49]. Insulin resistance induced by obesity plays a crucial role in elevated blood glucose levels. A low-glucose diet has been shown to improve insulin resistance in feasibility trials, suggesting its potential as a preventative measure against BC in postmenopausal women [50]. Circular RNAs (circRNAs), a member of the RNA family, regulates complex functional processes. Huang et al. reported that circKIF4 was overexpressed in BC cell lines and tissues, and inhibition of its expression suppressed BC growth and metastasis. CircKIF4 is involved in glucose metabolic reprogramming of BC, and silencing of circKIF4A dramatically affects glucose uptake and lactic acid production of BC cells, and it affects the metabolic reprogramming of BC mainly through the circKIF4A–miR-335–OCT4/ALDOA–HK2/PKM2 axis [51]. Tumour microenvironment is an important factor influencing BC growth and metastasis. Li et al. reported that GPR81-mediated reprogramming of glucose metabolism is associated with immune landscape in the tumour microenvironment of BC. G protein-coupled receptor 81 (GPR81) was significantly correlated with the glycolytic capacity of BC cells (P < 0.001), which depended on the Hippo-YAP signalling pathway (P < 0.001). These alterations in glycolytic capacity resulted in a decrease in the percentage of lymphocytes CD8^+^ T cells (P < 0.001) and an increase in the percentage of FOXP3^+^ T cells (P < 0.001) in the transwell co-culture system [52] (Fig. 3).

Alterations in glucose metabolism in BC, such as reprogramming of glucose metabolism, should be further investigated as a means of enriching the pathophysiological changes during BC development.

### Obesity-induced disorders of multiple cells and breast cancer

BC is not only correlated with metabolic disorders caused by obesity, but also with obesity-induced disorders of multiple cell types. Lipid-associated macrophages (LAMs) have been implicated in adversely affecting BC progression, correlating with the malignancy rate of the disease [53, 54]. It has been identified that STAB1 + TREM2 high lipid-associated macrophages exert immunosuppressive and pro-tumourigenic influences in TNBC [53]. The tumour microenvironment is widely mentioned in the study of solid cancers. It mainly includes epithelial cells, endothelial cells, blood and lymphatic vessels, bone marrow and lymphatic components present in the tumour. The complex network composed of the above components is called the tumour microenvironment [55]. Obesity, as a risk factor for breast cancer, has been reported to alter the breast cancer microenvironment by inducing changes in immune cells within the tumour microenvironment in a mice study using a high-fat diet [56]. Within the tumour-adipose microenvironment (TAME), lipid-associated macrophages represent a distinct macrophage subpopulation. The attenuation of LAMs has been reported to synergise with the antitumour effects of anti-PD1 therapy [54], positioning them as a novel subgroup for potential therapeutic targeting in BC management. Consequently, the tumour-adipose microenvironment, influenced by tumour-associated macrophages (TAMs), plays a pivotal role in cancer progression. Metabolic reprogramming plays a key role in T cell exhaustion in BC. In a mouse model of BC, a high-fat diet can promote the conversion of PD-1^−^CD8^+^ non-exhausted T cells into PD-1^+^CD8^+^ exhausted T cells, thereby promoting tumour development [57]. Single-cell RNA sequencing also revealed unique cellular expression in the tumour microenvironment of obesity-associated breast cancer. The report showed that obesity-associated epithelial cells in the tumour microenvironment of obese breast cancer patients had specific expression characteristics and were associated with tumour growth and hormone metabolism in breast cancer. Obesity-associated macrophages can also increase the proliferation of breast cancer tumour cells through transcription factors [58]. Obesity can also play a role in immune reprogramming through immune checkpoint blockade. In a mice experiment, BC was found to make the systemic immune system highly sensitive to obesity, resulting in greater immunosuppression. This immunosuppression can be alleviated by anti-PD-1 therapy [59]. Adipose-derived mesenchymal and stem cells have been shown to influence the tumour microenvironment and contribute to breast cancer progression and metastasis by releasing various cytokines and inflammatory mediators [60]. The enrichment of cancer stem cells (CSCs) within tumours is often driven by upregulation of IL-6 secretion via Stat3 pathway [8]. The pro-inflammatory factors including IL-6, IL-1β, and TNF-α notably increase in TAMs [61]. Depleting M2-like macrophages within this microenvironment has been shown to enhance the effects of anti-PD-1 therapy [54] (Fig. 3).

BC often emerges close to adipose tissue, which is abundant in the breast area. This proximity leads to the metabolic remodelling of adipocytes by BC cells, converting them into cancer-associated adipocytes (CAAs) [62]. CAA was demonstrated to be strongly associated with pro-inflammatory effects and cancer metastasis in the study [63]. In ER^+^ BC patients, a small number of adipocytes can promote the proliferation of a large number of BC cells [64]. These CAAs facilitate tumour angiogenesis and epithelial changes through secreting chemokines, such as chemokine (C–C motif) ligand 5 (CCL5) and chemokine (C–C motif) ligand 2 (CCL2), alongside with inflammatory mediators including IL-6 and TNF-α, which push BC cells towards a more aggressive phenotype [65] (Fig. 3). Furthermore, CAAs are implicated in the promotion of BC invasion and metastasis through a positive feedback mechanism involving Leukemia Inhibitory Factor (LIF)/chemokine (C–X–C motif) ligands (CXCLs). This process is primarily mediated by the binding of BC-derived CSCL1-3 and CSCL8 to the CSCR2 receptor, consequently activating the extracellular regulated protein kinases (ERK)1/2 pathway and the transcription factors NF-κB and Stat3 in a paracrine fashion, leading to the initiation of LIF expression, which in turn upregulates CXCLs [66]. This feedback loop elucidates the role of CAAs in enhancing BC invasion and metastasis, offering potential therapeutic targets.

A critical mechanism through which CAAs impact BC involves the influx of free fatty acids into BC cells [67]. As is mentioned, fatty acids are known to facilitate the proliferation and migration of BC via increased carnitine palmitoyltransferase 1A (CPT1A) and electron transport chain complex protein levels [68]. It has been reported that the co-culture of human adipocytes and BC cells can increase the expression of CD36 and fatty acid binding protein (FABP4). Of these, CD36 expression has been shown to lead to metabolic reprogramming and a shift towards fatty acid oxidation. In addition, CD36 has been found to directly interact with FABP4 to regulate fatty acid uptake, transport, and metabolism. The findings indicate that CD36 plays a pivotal role in facilitating the transfer of fatty acids from adipocytes to cancer cells and activating signalling pathways that promote [69].

Obesity can also lead to white adipose tissue (WAT) dysfunction [70]. WAT has been reported to be one of the factors that worsen the prognosis of BC. In patients with inflammation of mammary WAT, insulin, glucose, leptin, triglycerides, C-reactive protein and IL6 are increased, while high-density lipoprotein, cholesterol and adiponectin are decreased (P < 0.05) [71]. This indicates that WAT dysfunction is one of the factors affecting metabolism in obese individuals.

According to Adams, BD and others, 8 BMI-related miRNAs, including miR-191-5p and miR-122-5p, were found in the serum miRNA expression levels of 121 BC survivors who participated in hormone and physical activity experiments. This shows that weight gain caused by obesity can affect the levels of some miRNAs [72]. Recent studies have shown that inhibition of miR-191-5p inhibits BC cell growth and metastasis. MiR-191-5p can be directly transferred to macrophages, promoting macrophage M2-like polarisation and positive feedback to promote BC cell migration and invasion [73]. In addition, miR-122 has been reported to promote BC metastasis by reprogramming glucose metabolism [74]. In the analysis of the expression levels of lncRNAs related to BC (including GAS5 and LSINCT5), the expression levels of these two genes were lower in the overweight and obese group (BMI ≥ 25) than in the normal BMI group (BMI < 25). This tentatively explains the mechanism by which obesity affects BC through lncRNAs [75]. However, although the role of miRNAs and lncRNAs in BC has been widely demonstrated, no research has yet investigated the specific molecular mechanisms by which obesity affects BC through miRNAs and lncRNAs. Therefore, further experiments are needed to elucidate this process.

In summary, with more research, multiple cellular disorders caused by obesity may contribute to the increased risk of obesity-induced BC. In particular, the special changes in the tumour microenvironment and macrophage phenotypic transformation caused by obesity have certain research value.

This figure shows the interaction between BC cells and immune and metabolic regulatory factors in the tumour microenvironment. Cancer-associated adipocytes secrete the chemokines CCL2/5 and IL-6/TNF-α, which promote tumour proliferation and immune escape by binding to CXCR2 and IL-6R/TNF-R on the surface of tumour cells. At the same time, GPR81 upregulation can decrease CD8⁺ T cells and increase FOXP3⁺T cells through glucose metabolism reprogramming. M2-like macrophages can promote the effect of anti-PD-1 therapy on BC cells.

### Obesity-induced dysfunction of adipokines and breast cancer

It has been proved that adipokines play crucial roles in maintaining homeostasis and normal metabolic processes within the body [76]. Alterations in adipokine levels due to obesity significantly disrupt these physiological balances and have different functions in BC (Table 1).

**Table 1:**
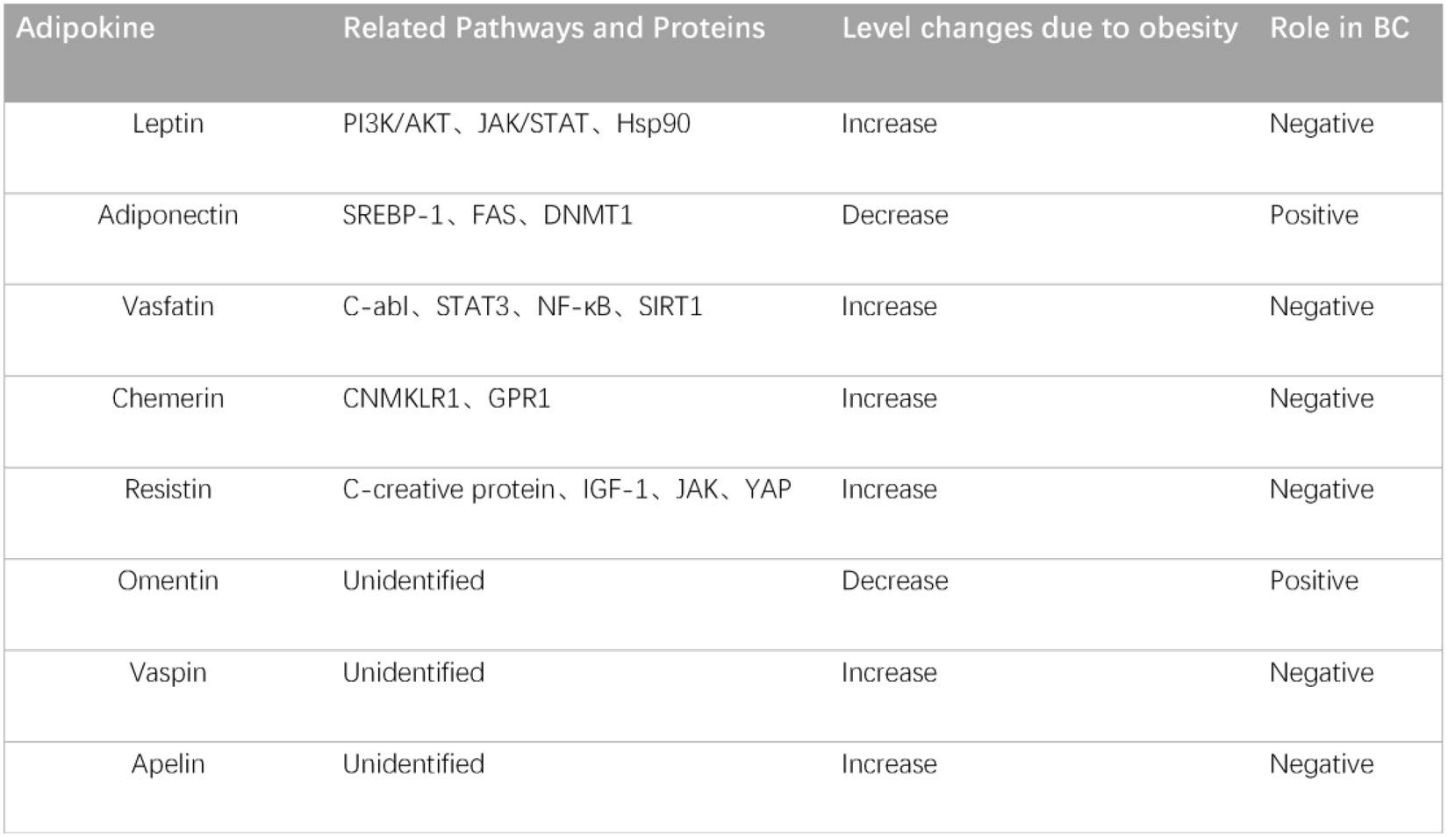
Role of obesity-associated adipokines in breast cancer

#### Leptin

Metabolic dysregulation within tumour-associated adipose tissue results in abnormal adipokine levels [77]. Hyperleptinemia is a high level of adipokines leptin. Hyperleptinemia profoundly influences every facet of BC, including onset and prognosis of disease, as well as its response to treatment [78]. Yuan et al. reported that elevated leptin levels increased phosphorylation of extracellular regulated protein kinases (ERK) in MCF-7 cells, thereby increasing proliferation and migration of MCF-7 human BC cells [79]. It has also been suggested that leptin-induced BC cell migration and ERK activation are mediated through autophagy, and that inhibition of autophagy reduced leptin-induced MCF-7 cell proliferation [80]. In TNBC, researchers found that baicalein can inhibit the expression of PD-L1 in the tumour environment by inhibiting leptin transcription from adipocytes. This enhances the anti-tumour immune response [81]. Meanwhile, leptin production by obesity-altered adipose stem cells has been reported to promote metastasis in TNBC [82]. Leptin signalling has been reported to increase the expression of genes associated with tumourigenesis and chemoresistance (*ABCB1*, *WNT4, ADHFE1, TBC1D3, LL22NC03, RDH5,* and *ITGB3*) in TNBC cells and to reduce the efficacy of chemotherapy [83]. Tamoxifen, a standard treatment for ER^+^ BC, demonstrates limited efficacy against other BC subtypes. Recent research indicates that elevated leptin levels can foster the proliferation of BC cells, even in the presence of tamoxifen [84]. Dashti et al. reported that high leptin concentrations are linked to an increased occurrence of ER positive BC [85]. Another study has also shown that hyperleptinemia is linked to increased occurrence of ER^+^ BC [86]. Chen et al. reported that celastrol significantly reversed leptin-triggered proliferation and metastasis of MCF-7 cells, which was mainly mediated by binding to the leptin receptor and controlling the downregulation of the PI3K/AKT pathway [87] . Leptin also mediates its effects on BC through microRNAs. Li et al. reported that adipocyte-derived leptin can promote PAI-1-mediated BC metastasis in a STAT3/miR-34a-dependent manner [88]. Remarkably, stable knockdown of ObR tumour switching to a less aggressive phenotype in ERα + and ERα-BC cells, where the absence of ObR reduced macrophage recruitment and also affected their cytokine mRNA expression profile [89]. In MCF-7/HER2 BC cells, leptin can render HER2-overexpressing cells resistant to tamoxifen by acting synergistically with the leptin/Ob–Rb/STAT3 pathway and the HER2 receptor to differentially regulate apoptosis-related genes [90].

Research has shown that leptin is closely associated with the development and metastasis of MCF-7 BC and ER ( +) BC. It is also associated with drug resistance in HER2-positive BC and TNBC. Although leptin has been widely studied as an earlier discovered adipokine, its role in the link between obesity and BC cannot be ignored.

#### Adiponectin

Obesity is associated with a reduction in adiponectin levels [91], a hormone known to exert anti-inflammatory, insulin-sensitising, and anti-atherosclerotic effects [92]. The up-regulation of pro-inflammatory factors due to obesity triggers the expression and enzymatic activity of DNA methyltransferase (DNMT1), which in turn suppresses adiponectin production [93]. Reports suggest that this substance may inducing apoptosis in BC cells by reprogramming fatty acid metabolism, predominantly through the suppression of lipogenesis by inhibiting the expression of sterol regulatory element-binding protein 1 (SREBP-1) and its target genes in the de novo FAS pathway. In addition, it appears to promote fatty acid degradation via fatty acid oxidation [94]. Moreover, globular adiponectin can inhibit BC cell proliferation by reducing the production of inflammatory vesicles [95]. Despite the mechanisms of adiponectin are not fully elucidated, numerous studies have identified a negative correlation between adiponectin levels and BC incidence, supporting the hypothesis that enhancing adiponectin levels could improve BC prognosis [96]. Interestingly, adiponectin has an opposite effect in ERα-positive and ERα-negative BC: it actually stimulates the growth of ERα-positive MCF-7 cells and inhibits the proliferation of ERα-MDA-MB-231 cells [97].

As an adipokine plays a protective role, the role of adiponectin in the link between obesity and BC deserves to be investigated.

#### Visfatin

Visfatin is originally identified as a B-cell differentiation factor, and it has been implicated in fostering tumour growth through various molecular mechanisms [98]. In existing studies, visfatin has been shown to induce proliferation of MCF-7 and MDA-MB-231 cells. It also improves cell viability and prevents TNF-α-induced apoptosis and PARP cleavage [99]. Another study showed that visfatin mainly promotes BC growth and metastasis by activating the c-Abl and STAT3 pathways [100]. An earlier study also highlighted that visfatin upregulates Notch 1 via NF-κB signalling, subsequently stimulating tumour growth [101]. Recent findings suggest that visfatin can drive the differentiation of monocytes into M2-like tumour-associated lymphocytes by inducing CXCL1 [102], with M2-like macrophages playing a crucial role in escalating tumour malignancy rates.

Furthermore, visfatin impacts BC progression through its interaction with adipose-derived stem cells (ADSCs). It activates the GDF15–AKT pathway in ADSCs, thereby encouraging tumour proliferation [103]. Visfatin enhances the expression of stemness-associated proteins and promotes angiogenesis by activating the SIRT1–SOX2 axis in BC [104]. Interestingly, post-surgical treatment of BC has been shown to reduce serum levels of visfatin in patients, suggesting that visfatin could potentially serve as a marker for Improving the prognosis of BC patients [105].

In summary, what makes visfatin special, as opposed to other adipokines, is the way it affects BC. Visfatin affects the development of BC by acting on immune-related pathways, causing inflammation and changes in immune-related cells. Therefore, if research on the association between obesity and BC is to be conducted from the immune aspect, visfatin will be a promising choice.

#### Chemerin

Chemerin is believed to have a similar protective role with adiponectin in various tumours, although it reportedly exhibits a tumour-promoting effect in gastric cancer [106]. This discrepancy raises the hypothesis that chemerin may act as a tumour suppressor through its binding to chemokine-like receptor 1 (CMKLR1) and G protein-coupled receptor 1 (GPR1) while potentially facilitating tumour progression by activating vascular endothelial growth [106]. The role of chemerin in promoting tumour vascularisation has been demonstrated in patients with squamous cell carcinoma of the oral tongue [107]. However, its role in BC has been less clearly defined.

Recent studies have identified NK cells and CD8^+^ T cells as key leukocytes mediating the effects of chemerin, with an observed absence of chemokine expression in BC cells suggesting a potential immune evasion mechanism [108]. Based on this, enhancing chemerin expression to attract immune effector cells into the tumour microenvironment and inhibit tumour growth presents a promising therapeutic strategy. This approach has already shown effectiveness in preventing bone metastases in BC [109].

The current understanding of chemerin’s role in BC is limited to a small body of research. Although a potential association between elevated serum chemerin levels and the prognosis of BC has been reported [110]. However, according to Akin et al., serum chemerin levels were not associated with BC stage [111]. While Song et al. suggested that it may be due to the small clinical sample size and lack of controls, Song et al. found that serum chemerin levels were associated with Ki67 expression levels and histology in BC, and that the combination with cancer antigen 15–3 (CA15-3) had relatively good diagnostic performance in BC [112].

In any case, the association between chemerin and BC and the specific mechanism of action remain to be explored, and there are currently few findings on its role as an adipokine in BC.

#### Other adipokines in breast cancer

Omentin has been identified as a potential marker for BC [113]. Cross-sectional studies, albeit with modest sample sizes, have found a negative correlation between circulating levels of Omentin-1 and postmenopausal BC [114]. However, the exploration of Omentin’s relationship with BC has been limited, with only a few studies investigating this link and involving small sample sizes.

Vaspin, a newly discovered adipokine, has been shown to improve insulin sensitivity mainly in white adipose tissue [115]. Studies have shown that Vaspin is positively correlated with BMI and can inhibit the expression of miR-33a-5p, thereby significantly enhancing the proliferation, invasion and metastasis of TNBC cells. Therefore, targeted therapy against Vaspin in patients with TNBC has become a potential therapeutic avenue [116]. Although low levels of vaspin have been associated with an increased risk of endometrial cancer, progress in understanding vaspin’s relationship with BC remains limited [117].

Apelin has also been linked to the development of TNBC in the context of obesity. Emerging research has confirmed that obesity-induced increases in apelin expression contribute to the progression of BC [118]. In another investigation, it was also noted that apelin mRNA expression was increased in subcutaneous adipose tissue as well as in TNBC in mice, and that increased apelin expression associated with increased levels of obesity in mice led to increased growth and brain metastasis of TNBC [119]. Insulin is the primary inducer of apelin, and intravenous apelin administration exerts a robust hypoglycemic effect under normal circumstances, apelin is mainly used to improve sugar absorption and reduce insulin resistance [120]. In contrast, obesity-induced insulin resistance is likely to be responsible for the marked increase in apelin levels in obese individuals. In addition, in another study, apelin was found to cause adverse reactions to neoadjuvant chemotherapy in BC patients [121]. However, it remains uncertain whether high apelin expression in tumours is directly associated with obesity.

The roles of Omentin, vaspin, and apelin in BC, as summarised from the limited research available, highlight the need for further studies to elucidate the specific contributions of these adipokines to BC, particularly in the context of obesity’s impact on the disease.

The table summarises the relevant molecules identified in existing reports on adipokines, as well as the level of change in adipokines in the serum of BC patients and their role in promoting or protecting against BC.

References: Leptin [87–89] Adiponectin [93, 94] Visfatin [100, 101, 104] Chemerin [106 Resistin^122^, ^123^] Omentin [113] Vaspin [115] Apelin [118]

### Obesity-induced inflammation and breast cancer

Adipocytes play a pivotal role in mediating inflammatory responses through the secretion of adipokines, as well as directly releasing inflammatory factors or mediating pro-inflammatory responses via alternative pathways [124]. Table 2 shows the main ways in which obesity leads to inflammation. A characteristic feature of the chronic inflammation within adipose tissue is the formation of crown-like structures, which results from the accumulation of macrophages around necrotic or dying adipocytes. Research has highlighted that death of adipocytes triggers a pro-inflammatory response from adipose-derived macrophages, notably without significant recruitment of blood monocytes, contributing to the chronic inflammatory response linked to obesity [125]. This chronic inflammatory milieu is a crucial element in the development and metastasis of BC promoted by obesity. Beyond this mechanism, the inflammatory responses driven by adipose tissue-associated inflammatory mediators, such as IL-6, also exhibit a strong association with BC. Studies have demonstrated that increased physical activity correlates with beneficial changes in IL-6 levels (R2 = 0.18; P < 0.03), positively influencing survival rates in overweight BC patients [126]. In addition, in BC patients, cytokine secretion, including IL-6, IL-8, and monocyte chemotactic protein-1 (MCP-1), is significantly elevated in tumour-associated adipose tissue. This enhances the communication between adipocytes and BC cells, thereby facilitating the growth and metastasis of BC [127]. Hence, it is evident that pro-inflammatory cytokines secreted by adipose tissue represent a significant pathway through which obesity impacts BC.

**Table 2:**
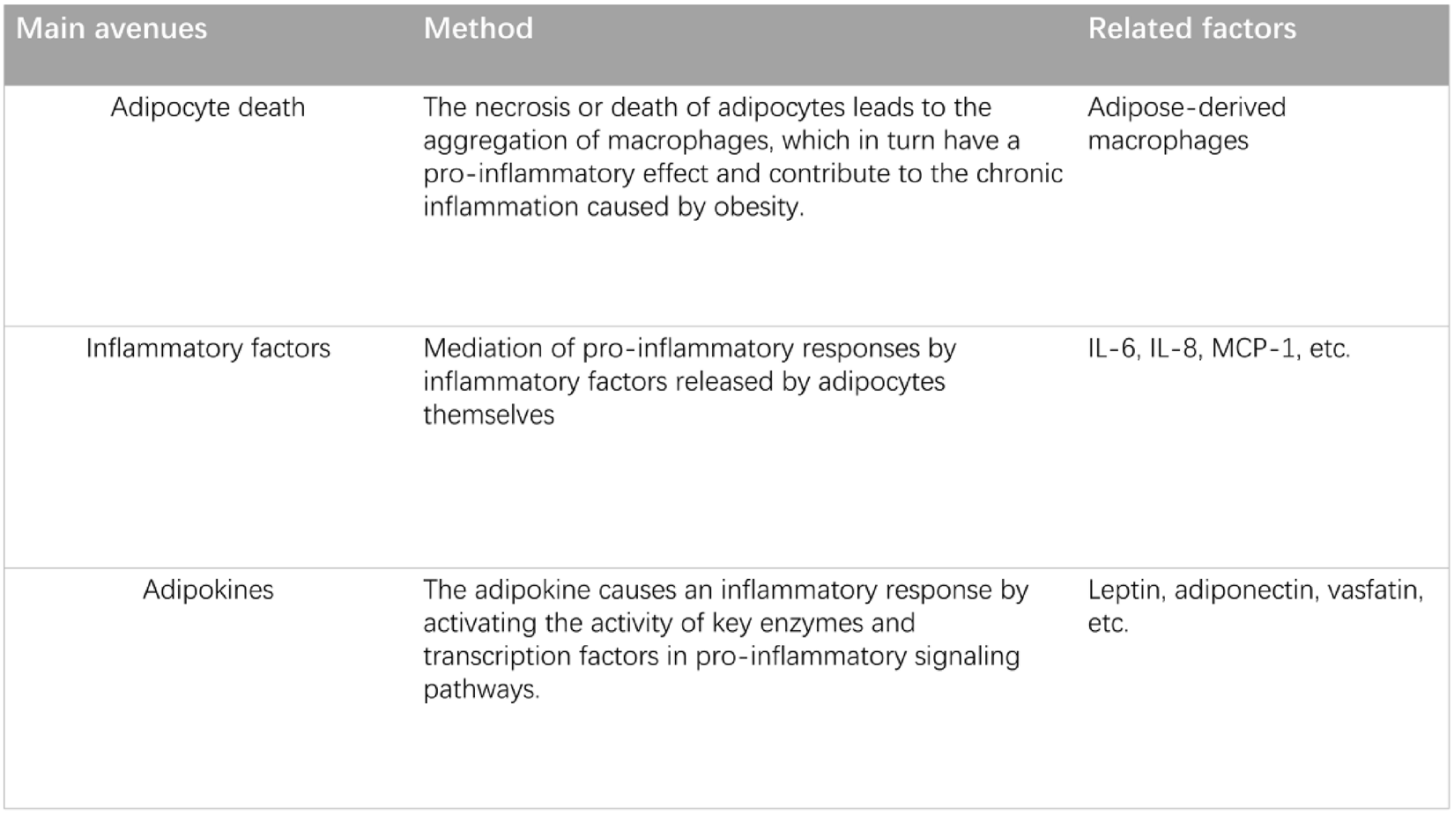
Main ways in which obesity leads to inflammation

In conclusion, adipose tissue contributes to the pro-inflammatory response through the necrosis of adipocytes and the secretion of cytokines. The subsequently pro-inflammatory state is a crucial link between adipose tissue and cancer cells, offering novel insights into potential therapeutic strategies, such as targeting IL-6, IL-8, and MCP-1 to disrupt the interaction between adipose tissue and tumour cells. However, further research is necessary to deepen our understanding of the crosstalk between tumour cells and adipocytes, aiming to identify more effective therapeutic targets.

The table summarises the three main pathways of obesity-induced inflammation reviewed in this article [125, 126]. In particular, adiponectin has anti-inflammatory and protective effects in BC [7, 71, 101, 102].

### Obesity-induced insulin resistance and breast cancer

Insulin resistance is identified as a significant factor that influences the incidence of BC, with elevated levels of insulin resistance being positively correlated with BC incidence in postmenopausal women [128]. Resistin, an adipokine composed mainly of 108 amino acids in humans and predominantly secreted by monocytes and macrophages, is a principal contributor to insulin resistance. It chiefly mediates insulin resistance in the hypothalamus by interacting with TOLL-like receptor 4 [122].

The exact relationship between insulin resistance and tumourigenesis remains unclear. Studies suggest a link between insulin resistance and inflammation. Heightened levels of insulin resistance are associated with elevated serum markers of inflammation, such as C-reactive protein, Interleukin-1β (IL-1β), IL-6, and TNF-α [129]. In macrophages deficient of glucocorticoid receptors, the pro-inflammatory response is amplified, further increases insulin resistance [130]. Study suggests that insulin resistance during treatment for advanced BC adversely affects recovery in patients receiving neoadjuvant chemotherapy. The chronic inflammation induced by obesity may play a pivotal role in the development of insulin resistance [131]. In addition, research into insulin resistance and CD8^+^ T cells has demonstrated that insulin resistance independently impacts CD8^+^ T cell activation [132], insights into potential immune-based strategies for BC treatment. Within the context of the insulin resistance pathway, insulin-like growth factor 1 (IGF-1) has been implicated in promoting angiogenesis in BC [133]. Investigation into the IGF-1/IGF-1R/FAK/YAP signalling pathway has revealed its role in facilitating the growth of TNBC [123]. This finding suggests a potential therapeutic avenue to mitigate BC malignancy and incidence by targeting the insulin-like growth factor 1 receptor (IGF-1R).

Insulin resistance has been implicated in both adipokines and inflammation, with leptin in particular dominating. Chronic inflammation caused by increased adipokines, in turn, exacerbates insulin resistance. Therefore, as an intermediate carrier of obesity in BC, it is of great significance to study insulin resistance to enrich its pathophysiological process.

## Treatment related to obese breast cancer patients

As obesity rates rise, the number of obese BC patients continues to increase. It has become a research priority to improve treatment strategies for obese BC patients.

### Weight loss therapy and breast cancer

Recent clinical studies have confirmed that bariatric therapy is effective in treating obese patients with BC [134]. Obesity usually leads to an increase in adipose tissue. Adipose tissue, as a complex endocrine organ, has already been mentioned in the previous section, where we have mentioned several ways in which it affects BC. Consequently, certain weight loss medications, such as orlistat, can serve as adjunct therapies for BC patients. Lipid metabolism plays a crucial role in the sustenance of BC cells, suggesting that strategies to inhibit lipid accumulation in adipocytes or cancer cells could provide novel approaches for BC therapy. For instance, orlistat has been shown to diminish the expression of the fatty acid-binding protein (FABP5) oncogene, thereby reducing lipid deposition and mitigating its tumourigenic effects [135]. Since Orlistat notably influences the metabolism of cancer cells by inducing hypolipidemic effects, it leads to notable antiproliferative, pro-apoptotic and anti-angiogenic actions [136]. Thus, orlistat presents a promising pharmacological option for the clinical management of BC.

Moreover, glucose-lowering medications like metformin have shown beneficial effects on BC. The administration of metformin in conjunction with BC therapy, there has been a noted decrease in the phosphorylation of PKB/Akt and ERK1/2, along with reductions in insulin levels and insulin receptor activity, aligning its anticancer properties [137]. In addition, metformin has been identified to impede metastasis and growth in HER-2 positive BC cell lines and mouse models, offering protective effects against the development of HER-2 positive BC. However, in clinical settings, its anticancer efficacy and impact on survival rates have not shown significance [138].

Moghadam, BH, et al. also found that when comparing inflammatory and physical indicators of obese BC survivors who did high-intensity intermittent training with those who did moderate-intensity continuous training, the high-intensity interval training group had greater changes in body weight, fat mass, TNF-α, and leptin levels, proving that high-intensity interval training may be a more ideal treatment option [139]. Therefore, losing weight through physical training can also achieve the effect of treating obese BC patients.

In an experiment in which mice underwent vertical sleeve gastrectomy, they found that weight loss by gastrectomy did not have an anti-immune checkpoint blockade effect, while mice that lost the same amount of weight by diet-induced weight loss were able to effectively counteract the tumour microenvironment and improve immunosuppression [140].

Diet control is also an important part of weight loss therapy. In a fasting mimicking study of 131 randomly assigned HER2-negative BC patients (NCT02126449) who received 3 days of either a fasting mimicking diet or a regular diet before and during chemotherapy, the results showed that the fasting mimicking diet significantly reduced chemotherapy-induced DNA damage in T lymphocytes [141]. In addition, in experiments with mice (NCT02126449), diets rich in specific phytochemicals have preventive and interventional value in BC [142]. A study of a long-term anti-inflammatory diet found a statistically significant association between an anti-inflammatory diet and the risk of death in BC survivors (NCT0169699). The diet improved survival in BC survivors [143]. A randomised controlled trial (NCT03045289) also reported that a plant-based diet improved weight, cardiometabolic and hormonal factors in patients with metastatic BC [144]. In another randomised clinical trial (NCT00003787) involving Chinese and American women, a soy diet was shown to reduce the risk of recurrence in BC patients [145] (Table 3). Therefore, die control is a promising area of research in BC treatment.

**Table 3:**
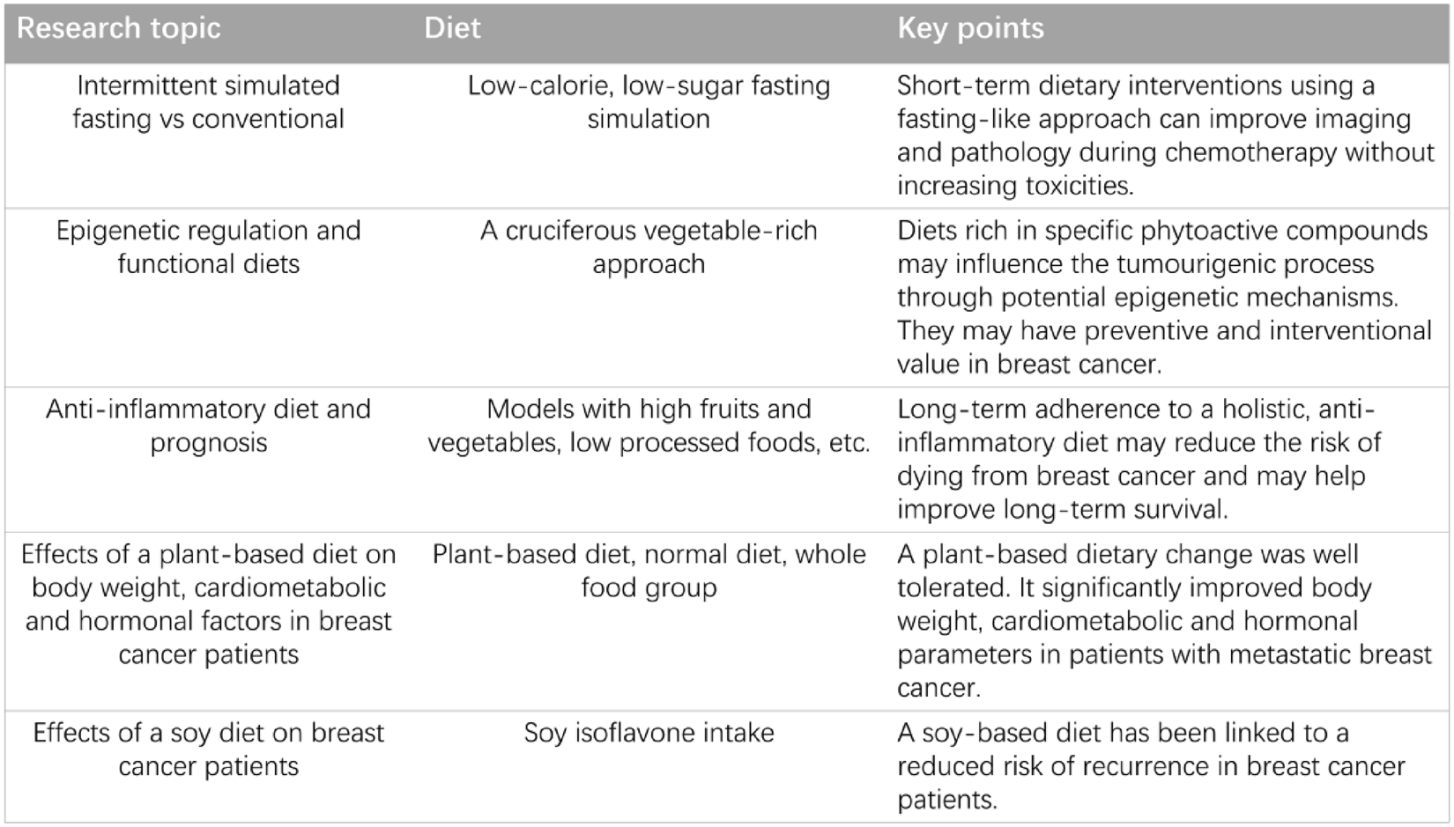
Studies of various diet controls in breast cancer

These insights underscore the potential of weight loss supplements as a component of BC treatment regimens, indicating a growing acceptance of such interventions. Nonetheless, additional research is imperative to confirm their clinical effectiveness and to refine therapeutic strategies for BC.

This table summarises studies that have investigated the effects of dietary changes on BC, such as fasting simulations, plant-based diets, and soy diets.

References: [141–145]

### Relevant therapeutic advances and breast cancer

Studies have revealed that miR-1304-3p can activate CAAs, stimulate lipid release, and enhance cancer cell growth. This microRNA is notably overexpressed in African Americans and offers insights into treatment approaches for this demographic while highlighting the association between exosomes and CAAs [146]. In addition, in BC patients with type 2 diabetes, exosomes derived from CAAs have been reported to increase the aggressiveness of BC [147]. Recent breakthroughs in BC research have highlighted the therapeutic potential of targeting CAAs. BZ26, a derivative of the peroxisome proliferator-activated receptor γ (PPARγ) antagonist GW9662, can halt the proliferation and invasion of BC cells by inhibiting the reprogramming of mature adipocytes into CAA-like cells [148]. Considering the role of CAAs in promoting BC progression via continuous secretion of adipokines and inflammatory factors, strategies focused on curbing the formation of CAAs emerge as a promising therapeutic approach.

These insights into the molecular actions of leptin not only suggest avenues for targeted molecular therapies but also offer strategies for improving patient outcomes, such as employing combination therapies with PD-L1/PD-1 and Arginase 1 (ARG1) antibodies specifically in obese patients, or modulating exosome production in BC cells by targeting the leptin/leptin receptor/Hsp90 axis [89, 149]. Leptin extends to influence macrophage activity within the tumour micro-environment. In contrast, lipid-associated macrophage depletion in the tumour microenvironment synergises with the anti-tumour effects of anti-PD-1 therapy, so leptin was used to modulate macrophage activity in the tumour microenvironment to achieve improved therapeutic efficacy would be a viable option [54, 89, 149]. Notably, post-recovery serum concentrations of leptin may serve as a prognostic marker for assessing recurrence risk in BC survivors [150]. In addition, strategies to restore or elevate adiponectin levels have emerged as a focal point of recent research. Studies on the adiponectin receptor agonist therapy, such as liraglutide, have demonstrated antiproliferative effects in various cancers including prostate, pancreatic, and hepatocellular cancers [151].

In immunotherapy for obese and normal weight BC patients. Obesity has been reported to cause resistance to immunotherapy in BC patients, among BC patients undergoing chemotherapy, obese BC patients have a significantly lower density of tumour-infiltrating lymphocytes than those of normal weight and those who are overweight [152]. Obesity can promote immunotherapy resistance in obese mice by increasing the levels of CXCL1 in the BC microenvironment and over-activating CD8 tumour-infiltrating lymphocytes [153].

FASN inhibitors are commonly used in targeted therapies for BC. When the FASN inhibitor TVB-3166 was used to treat MCF-7 BC cells, BC cell survival and proliferation were decreased, and apoptosis was increased in tumour cells treated with serum exposure (BMI greater than 30). These results indicate that FASN is a potential therapeutic target for obesity-induced BC invasion [154]. A new generation of FASN inhibitors, such as TVB-2640, has been shown in recent experiments to have a synergistic effect with the topoisomerase inhibitor SN-38. They upregulate fatty acid synthase, downregulate the expression of cell cycle progression genes and slow the motility of TNBC brain metastasis cell lines [155]. Targeted therapies other than FASN inhibitors have also been reported. For example, the CDK4/6 inhibitor palbociclib has been shown to have an increased incidence and severity of side effects in obese and overweight BC patients treated with palbociclib, as well as an increased rate of treatment discontinuation [156]. In addition, in a study of another CDK4 inhibitor, abemaciclib, oral abemaciclib at a dose approved for use in humans maintained the amount of fat in diet-induced obese mice by increasing lipid oxidation without a significant reduction in lean body mass [157].

As mentioned earlier, reprogramming of glucose metabolism is one of the important characteristics of BC cells. Recently, some studies have shown that a type of metal–organic framework nanoparticles coated with macrophage membranes can regulate glucose metabolism for the treatment of TNBC. These nanoparticles can, through carefully planned interventions, stimulate the accumulation of lactic acid in tumour cells and induce apparent intra-tumour acidosis, providing a way to treat tTNBC by manipulating glucose metabolism [158]. In another study, the branched-chain alpha-keto acid dehydrogenase kinase inhibitor 3,6-dichlorobenzo [b]thiophene-2-carboxylic acid showed antitumour effects in patient-derived tumour xenograft models, providing potential progress in the treatment of TNBC through glucose metabolic reprogramming [159].

Obesity is often a major risk factor for type 2 diabetes [160]. Type 2 diabetes has been reported in studies as an independent factor for poorer prognosis of BC [161]. Diabetes is a chronic inflammatory disease. In a study constructing an lncRNA–mRNA co-expression network, it was found that lncRNAs and mRNAs are highly interconnected and form co-expression networks associated with diabetes inflammation [162]. In another experiment in C57BL/6 mice, the researchers showed that this chronic inflammatory state may be mediated by the PI3K/AKT pathway [163].

Metformin, a drug used to treat type 2 diabetes, has been reported in epidemiological evidence to have a preventive effect on BC [164]. However, subsequent research has shown the opposite. In randomised clinical trials of metformin, metformin did not improve survival in BC patients compared with placebo [165]. In addition, another drug, glucagon-like peptide-1 receptor agonist, which has FDA approval for the treatment of obesity, has been found to potentially promote the malignant progression of TNBC [166].

It is worth noting that radiotherapy and chemotherapy for breast cancer can induce changes in the mRNA levels of renin–angiotensin-related genes in cardiac tissue, which may be associated with cardiac toxicity induced by breast cancer treatment [167]. In another clinical observational study, it was found that antihypertensive drugs such as beta-blockers can reduce the risk of breast cancer recurrence [168].

## Conclusion and prospect

The complexity of the relationship between obesity and BC lies in the fact that it involves not only metabolic imbalances and changes in the immune microenvironment, but also the combined effects of multiple factors. These include remodelling of lipid and glucose metabolism, abnormalities of various adipokines, low-grade chronic inflammation and insulin resistance.

The current understanding of how obesity affects BC has expanded from a simple energy surplus to include multiple links, such as fatty acid synthesis pathways, cholesterol metabolic reprogramming, the immune microenvironment and inflammatory pathways. However, these molecular pathways are not isolated from each other. Further research is needed to elucidate their interactions, causal sequences and how these alterations play different roles in different subtypes of BC in the context of integrating multiple pathways, laying the foundation for precision intervention. At the same time, there is much to be learned about the interaction between dysregulated adipokine levels and different immune cell types and the tumour microenvironment. Increased research into how adipokines regulate the recruitment, differentiation and function of immune cells may help us gain a deeper understanding of the mechanisms of immune escape and BC progression in the context of obesity. Obesity-related BC is characterised by increased glycolysis, fatty acid synthesis and activation of cholesterol metabolic pathways. In addition, chronic inflammation and increased inflammatory factors play an important role in tumourigenesis and drug resistance. In the future, combined blockade of key metabolic enzymes, lipid accumulation pathways or inflammatory signalling nodes may be used to explore personalised treatment strategies for patients with obesity-related BC. Similarly, reducing obesity-induced drug resistance is an important direction. 

In conclusion, to achieve personalised and precise prevention and treatment of obesity-related BC, future research and clinical application should urgently investigate how obesity affects BC development and progression from multiple perspectives, further analyse the underlying mechanisms of adipokines, chronic inflammation and metabolic reprogramming, and explore reliable new diagnostic and therapeutic strategies based on these mechanisms of action.

## Data Availability

No datasets were generated or analysed during the current study.

## Abbreviations

BC: Breast cancer
TNBC: Triple negative breast cancer
BMI: Body mass index
SNPs: Single-nucleotide polymorphisms
*FTO*: Obesity-associated gene
*MC4R*: Melanocortin 4 receptor gene
IL-6: Interleukin-6
IL-8: Interleukin-8
TNF-α: Tumour necrosis factor-α
ER +: Estrogen receptor-positive
PR +: Progesterone receptor-positive
FLI: Fatty liver index
LDL: Low-density lipoprotein
NSDHL: NAD/NADP-dependent steroid dehydrogenase-like
ROS: Reactive oxygen species
ERRα: Estrogen-related receptor α
FASN: Fatty acid synthase
Elovl5: Very long-chain fatty acid protein 5
TGF-β: Transforming growth factor-β
LD: Lipid droplet
HER2: Human epidermal growth factor receptor 2
circRNAs: Circular RNAs
GPR81: G protein-coupled receptor 81
LAMs: Lipid-associated macrophages
TAME: Tumour-adipose microenvironment
CAAs: Cancer-associated adipocytes
CCL5: Chemokine (C–C motif) ligand 5
CCL2: Chemokine (C–C motif) ligand 2
TAMs: Tumour-associated macrophages
CSCs: Cancer stem cells
LIF: Leukemia inhibitory factor
CXCLs: Chemokine (C–X–C motif) ligands
CPT1A: Carnitine palmitoyltransferase 1A
FABP4: Fatty acid binding protein
ERK: Extracellular regulated protein kinases
WAT: White adipose tissue
DNMT1: DNA methyltransferase
SREBP-1: Sterol regulatory element-binding protein 1
ADSCs: Adipose-derived stem cells
CMKLR1: Chemokine-like receptor 1
GPR1: G protein-coupled receptor 1
CA15-3: Cancer antigen 15–3
MCP-1: Monocyte chemotactic protein-1
IL-1β: Interleukin-1β
IGF-1: Insulin-like growth factor 1
IGF-1R: Insulin-like growth factor 1 receptor
FABP5: Fatty acid-binding protein
PPARγ: Peroxisome proliferator-activated receptor γ
ARG1: Arginase 1
